# Streptochlorin Suppresses Allergic Dermatitis and Mast Cell Activation via Regulation of Lyn/Fyn and Syk Signaling Pathways in Cellular and Mouse Models

**DOI:** 10.1371/journal.pone.0074194

**Published:** 2013-09-27

**Authors:** Seung-Hwan Lee, Hee Jae Shin, Dong-Young Kim, Do-Wan Shim, Tack-Joong Kim, Sang-Kyu Ye, Hyung-Sik Won, Sushruta Koppula, Tae-Bong Kang, Kwang-Ho Lee

**Affiliations:** 1 Department of Biotechnology, College of Biomedical and Health Science, Research Institute of Inflammatory Diseases, Konkuk University, Chungju, Korea; 2 Marine Natural Products Chemistry Laboratory, Korea Institute of Ocean Science and Technology, Ansan, Korea; 3 Division of Biological Science and Technology, Yonsei University, Wonju, Korea; 4 Department of Pharmacology, Seoul National University, College of Medicine, Seoul, Korea; Virginia Tech University, United States of America

## Abstract

Allergic diseases are chronic inflammatory conditions with specific immune and inflammatory mechanisms. Scientific interest in understanding the mechanisms and discovering novel agents for the prevention and treatment of allergic disease is increasing. Streptochlorin, a small compound derived from marine actinomycete possesses anti-angiogenic and anti-tumor activities. However, the anti-allergic effects and underlying mechanisms remain to be elucidated. In the present study, we investigated the effect of streptochlorin on allergic responses *in vitro* and *in vivo*. Streptochlorin inhibited degranulation and production of tumor necrosis factor-α and IL-4 by antigen-stimulated mast cells. Streptochlorin also inhibited the phosphorylation of Akt and the mitogen-activated protein kinases (MAPKs), including p38, ERK, and JNK. Further, streptochlorin reduced the phosphorylation of Syk in RBL-2H3 cells and inhibited the activity of Lyn and Fyn. Furthermore, administration of streptochlorin suppressed the allergic reactions in both passive cutaneous anaphylaxis reaction and 2, 4-dinitrofluorobenzene (DNFB)-induced allergic dermatitis in mice model. Considering the data obtained, we report for the first time that streptochlorin possess anti-allergic properties. The underlying mechanism of streptochlorin in exhibiting potent anti-allergic activity might be through the inhibition of the Lyn/Fyn and Syk signaling pathways.

## Introduction

Allergic diseases, including dermatitis, rhinitis, asthma, and eczema, can be triggered by a number of factors. The majority of these diseases are chronic relapsing inflammatory diseases that are associated with genetic predisposition, environmentally triggered cutaneous hyperreactivity and immune dysregulation [1]. Mast cells play a central role in allergic responses that are targeted by allergens, such as the skin, airway mucosa, gastrointestinal tract, adventitia of blood vessels, and the myocardium. In addition, aggregation of allergens, immunoglobulin E (IgE), and high-affinity IgE receptor (FcεRI) on mast cells initiates a degranulation-mediated allergic reactions and synthesis of lipid mediators to induce the early phase of allergic responses [2], [3], [4]. Therefore, inactivation of mast cells is a promising approach in preventing allergic diseases.

IgE bound FcεRI aggregation initiate antigen-dependent mast cell activation through regulation by a complex series of intra-cellular signaling processes. Initial signaling events from FcεRI occupation involve coalescence of the aggregated receptors with specialized micro domains of the plasma membrane known as lipid rafts, activation of Src-family kinases and subsequently, tyrosine phosphorylation of the receptor subunits [5]. The main Src-family kinase involved in these initial stages is Lyn [5]. When the tyrosine residues of FcεRI β-chain and γ-chain are phosphorylated by Lyn, phosphorylated immunoreceptor tyrosine-based activation motifs (ITAMs) of FcεRI β- and γ-chain provide high-affinity docking sites for SH2 domains of Lyn and ZAP70 (ζ-chain-associated protein kinase of 70 kDa)-related tyrosine kinase Syk (spleen tyrosine kinase). The subsequent Syk- and/or Lyn-mediated tyrosine phosphorylation of the transmembrane adaptor molecule LAT (linker for activation of T cells) is crucial for coordination of the downstream signaling pathways that are required for the release of the various pro-inflammatory mediators [6]. On the other side, following FcεRI aggregation, the protein tyrosine kinase Fyn becomes activated, which results in tyrosine phosphorylation of the cytosolic adaptor molecule GAB2 (growth-factorreceptor-bound protein 2 (GRB2)-associated binding protein 2). This leads to the binding of phosphatidylinositol 3-kinase (PI3K) by GAB2, resulting in an increase in calcium (Ca^2+^) mobilization, which potentially occurs through a mechanism that involves the BTK (Bruton's tyrosine kinase)-dependent phosphorylation of phospholipase Cγ (PLCγ) [5].

**Figure 1:**
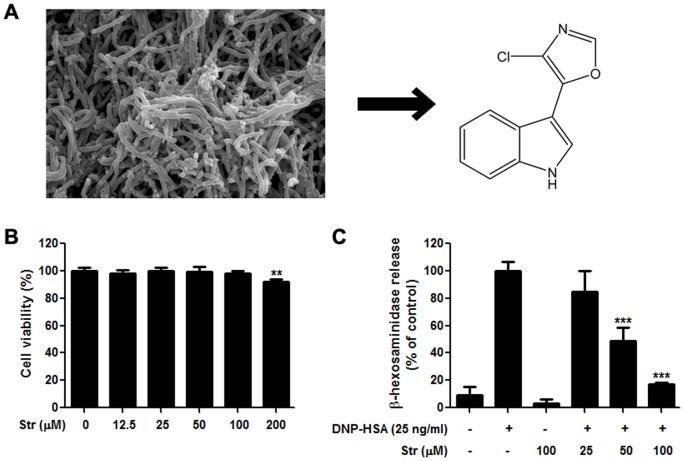
Effect of streptochlorin on cytotoxicity and degranulation in RBL-2H3 cells. (A) *Streptomyces* sp. (strain 04DH110) and its molecular structure. (B) Cell viability assay of indicated concentrations of streptochlorin in RBL-2H3 cells measured by MTT assay. (C) Degranulation was determined by measuring β-hexosaminidase activity in RBL-2H3 cells. The values indicate mean ± standard error of the mean (*n* = 3), from three independent experiments by one-way analysis of variance followed by Dunnett's multiple comparison test. ** *P*<0.01 and *** *P*<0.001 significant difference compared with the DNP-HSA only treated group. Str, streptochlorin; DNP-HSA, dinitrophenyl-human serum albumin.

Existing therapies using synthetic compounds in treating allergic diseases are largely ameliorative rather than curative and may further lead to unexpected side effects, such as alterations in gastric irritation, redistribution of fat and peptic ulcer formation [7]. Therefore natural products and their derived agents might be a good alternative approach. Mounting evidence suggested that the bioactive potential of marine microbes is amazingly diverse and productive. Studies have shown that bioactive compounds from marine microorganisms are not generally cytotoxic but rather are targeted to specific cellular events and therefore have great potential as anti-microbial, anti-cancer, or anti-inflammatory agents [8]–[10]. Streptochlorin, a yellowish amorphous solid compound isolated from *Streptomyces* sp. (Fig. 1A) possesses selective cytotoxicity against several cancer cell lines [11], [12]. Pharmacologically, streptochlorin possess anti-angiogenic and anti-tumor properties. In this study, we report the anti-allergic effects of streptochlorin and the underlying mechanisms involved in cellular and animal models of allergy in several aspects.

## Materials and Methods

### Ethics Statement

The experiments were conducted in accordance with the “Guide for the Care and Use of Laboratory Animals” adopted by the United States National Institutes of Health. The study protocol was reviewed and approved by the Committee on the Ethics of Animal Experiments of the Konkuk University (Permit Number: 08037).

### Reagents

Streptochlorin was obtained from the fermentation broth of marine *Streptomyces* sp. strain 04DH110 that was isolated from marine sediment collected at Ayajin, Korea. Minimum essential medium (MEM), fetal bovine serum (FBS), penicillin, and streptomycin were from Hyclone (Logan, UT, USA), and other cell culture supplies were from Becton Dickinson (Franklin Lakes, NJ, USA). TRIzol^TM^ reagent was from Molecular Research Center (Cincinnati, OH, USA), and oligo dT 18 primer, AccuPower^TM^ RT Premix, AccuPower^TM^ PCR Premix, and primers against tumor necrosis factor-α (TNF-α) and interleukin-4 (IL-4) were from Bioneer (Seoul, Korea). Dinitrophenyl-human serum albumin (DNP-HSA) and all other chemicals were from Sigma-Aldrich (St. Louis, MO, USA), and all antibodies, including phosphor-Syk (Tyr 525/Tyr 526) were from Cell Signaling Technology (Beverly, MA, USA).

### Cell Culture

The rat basophilic leukemia (RBL) 2H3 cell line was obtained from the American Type Culture Collection (ATCC, Manassas, VA, USA) and was maintained in MEM supplemented with 10% heat-inactivated FBS and antibiotics (100 U/mL of penicillin and 100 µg/mL of streptomycin) in a humidified atmosphere of 5% CO_2_ at 37°C.

### Viability Assay

Cell viability was determined using the 3-(4, 5-dimethylthiazol-2-yl)-2, 5-diphenyltetrazolium bromide (MTT) assay. RBL-2H3 cells were plated in triplicate at a concentration of 1×10^4^ cells per well in a 96-well plate and incubated for 7 h prior to treatment with various concentrations of streptochlorin. After treatment the medium was discarded and 100 µL of MEM containing 0.5 mg/mL MTT was added to each well. After incubation for 3 h at 37°C, the medium was discarded and 200 µL of dimethyl sulfoxide (DMSO) was added to dissolve the MTT formazan. The optical density was measured at 550 nm with a spectrophotometer.

### Measurement of β-hexosaminidase

Anti-DNP IgE-sensitized RBL-2H3 cells (2.5×10^5^ cells per well in a 24-well plate) were pretreated with various concentrations of streptochlorin for 1 h and stimulated in the absence or presence of DNP-HSA (25 ng/mL) for 20 min. The culture supernatant (30 µL) was mixed with 30 µL of substrate buffer (1 mM 4-p-nitrophenyl-N-acetyl-β-D-glucosaminidase in 0.1 M citrate buffer, pH 4.5) for 1 h. After incubation, 250 µL of 0.1 M sodium carbonate-bicarbonate buffer was added and the absorbance was measured at 405 nm.

### Measurement of Cytokines and IgE

Interferon-γ (IFN-γ), TNF-α, IL-4, and IgE were measured using a BD OptEIA^tm^Set enzyme-linked immunosorbent assay (ELISA) kit (BD Biosciences, San Jose, CA, USA) according to the manufacturer's instructions. The absorbance at 450 nm was determined using a microplate reader (Molecular Devices, Sunnyvale, CA, USA).

### RNA Isolation and Reverse Transcription-Polymerase Chain Reaction (RT-PCR)

Total RNA was isolated from RBL-2H3 cells with TRIzol reagent according to the manufacturer's instructions. Total RNA (2 µg) was reverse transcribed to make cDNA by using the oligo dT 18 primer and AccuPower™ RT Premix. Synthesis of cDNA was performed at 42°C for 60 min followed by inactivation at 94°C for 5 min. PCR amplification was conducted in a 20 µL reaction mixture with 0.2 µg of cDNA and 10 pM of each primer. PCR was performed using primers for TNF-α, IL-4, and β-actin. Analysis of the resulting PCR products on 1% agarose gels showed a single band of amplified DNA with the expected sizes.

### Immunoblotting

Cells were lysed in a buffer containing 50 mM Tris–HCl (pH 8.0), 1% Nonidet P-40 (NP-40), 150 mM sodium chloride, 0.5% sodium deoxycholate, 0.1% sodium dodecyl sulfate (SDS), protease and phosphatase inhibitor cocktails. Equivalent amounts (40 µg) of samples were resolved by 10% SDS-polyacrylamide gel electrophoresis (SDS-PAGE), and the separated proteins were transferred to a polyvinylidene fluoride membrane. The membrane was blocked for 1 h at room temperature with Tris-buffered saline (TBS) containing 5% fat-free dried milk. Next, the membrane was incubated overnight at 4°C with the primary antibody diluted 1∶1000–5000, and immune complexes were incubated for 1 h at room temperature with horseradish peroxidase-conjugated antibody diluted 1∶1000–5000. After application of the secondary antibody, the membrane was washed three times with TBS containing Tween-20. The reactive products were visualized by using an enhanced chemiluminescence detection kit and a model LAS-3000 luminescent image analyzer (Fujifilm, Tokyo, Japan).

### Flow Cytometry

The expression of FcεRI on the surface of RBL-2H3 cells was analyzed with a FACSCalibur apparatus (BD Biosciences). In brief, RBL-2H3 cells were harvested, washed, and suspended in cold phosphate-buffered saline (PBS) containing 1% FBS, 0.02% NaN_3_, and 0.053 mM EDTA. The cells were then incubated with anti-CD16/CD32 IgG to reduce nonspecific binding, followed by serial incubations with a saturating concentration of fluorescein isothiocyanate (FITC)-conjugated anti-FcεRI antibody for 1 h at 4°C. At least 25,000 stained cells were analyzed by FACSCalibur (BD Biosciences, San Jose, CA, USA).

### Lyn and Fyn Kinases Assay

Activity of Lyn and Fyn kinases *in vitro* was measured using the Kinase Enzyme System and ADP-Glo™ Kinase assay (Promega, Madison, WI, USA) according to the manufacturer's instructions. Briefly, in a 96-well plate (25 µL total reaction volume), 0.1 µg each of Lyn and Fyn was incubated in Reaction Buffer A (8 mM Tris, pH 7.5, 4 mM MgCl_2_, 0.02 mg/mL bovine serum albumin) supplemented with 50 µM dithiothreitol (DTT) for 10 min. Incubated kinases were mixed with 1 µg of each substrate peptide and 50 µM ATP, followed by incubation for 15 min. The mixtures were incubated at room temperature for 40 and 30 min after addition of ADP-Glo™ Reagent and Kinase Detection Reagent, respectively. Luminescence was detected using a SpectraMax L apparatus (Molecular Devices) at a wavelength of 470 nm.

### Induction of IgE-Mediated Passive Cutaneous Anaphylaxis (PCA) in Mouse

Female, 6-week-old, Balb/c mice were purchased from Nara Biotech (Seoul, Korea) and housed under specific pathogen-free (SPF) conditions at Konkuk University animal care facility. Mice were passively sensitized by intradermal injection in the ears with 500 ng monoclonal mouse DNP-specific (anti-DNP) IgE in 30 µL of PBS. Streptochlorin (1 and 5 mg/kg) was intravenously injected 23 h after anti-DNP IgE injection. After 1 h, mice were intravenously injected with 100 µg of DNP-HSA in 200 µL PBS containing 1.5% Evans blue via tail vein, and mice were sacrificed after 20 min. The Evans blue was extracted by incubating the same size ears in 200 µL formamide for 12 h at 64°C. The absorbance of Evans blue was measured at 650 nm.

### DNFB-Induced Allergic Dermatitis

Twenty-five microliters of 0.15% 2, 4-dinitrofluorobenzene (DNFB) in acetone–olive oil (3∶1) was applied to each mouse ear lobe (6-week-old Balb/c mice) once every week for 5 weeks. Streptochlorin was injected with saline into the peritoneal cavity once every 3 days. Ear thickness over the time course of the experiment was determined with a digital caliper (Control Company, Friendswood, TX, USA).

### Myeloperoxidase (MPO) Assay

Hydrochloric acid (34 µL, 3.7%) was added to 5 mg of 3, 3′, 5, 5′-tetramethylbenzidine (TMB), followed by 1 mL of DMSO. This stock solution was slowly added to a sodium acetate–citric acid buffer (0.1 mol/L, pH 6.0) at a ratio of 1∶100. TMB solution (100 µL), 10 µL of homogenized sample, and 25 µL of 1 mM hydrogen peroxide (H_2_O_2_) were added to a microtiter plate and allowed to react for 30 min. The reaction was stopped with 100 µL of 1 N H_2_SO_4_. Changes in optical density were monitored at 450 nm.

### Histology

For histological analysis, ear lobes were embedded in paraffin and sliced in 6 µM thick sections with a microtome (Leica Microsystems, Jena, Germany). Before staining, sections were deparaffinized with xylene. Sections were stained with hematoxylin (Merck, Whitehouse Station, NY, USA) and 0.5% eosin (Sigma-Aldrich) to observe morphologic changes and cell infiltration. Toluidine blue (pH 3.0) was used to detect mast cells.

### Analysis of T-cell division with CFSE

For measurement of CFSE-incorporated proliferation, inguinal lymph node cells were-incubated with CFSE (5 μM; Sigma-Aldrich, St. Louis, MO, USA) in PBS for 8 min at room temperature, washed with RPMI 1640 media, pretreated with streptochlorin at indicated concentrations, and then incubated for 48 h on 96-well plates coated with anti-CD3/anti-CD28 (both eBioscience, San Diego, CA, USA). Cells were stained with PE-labeled anti-Thy1 to distinguish T cells. The cells were analyzed by FACSCalibur.

### Statistical Analysis

Data were expressed as mean ± standard error of the mean of at least three separate experiments conducted in triplicate. Statistical comparisons between all groups were performed by using one-way analysis of variance (ANOVA) followed by the Dunnett's test. *P* values <0.05 were considered statistically significant.

## Results

### Streptochlorin inhibits antigen-induced degranulation in RBL-2H3 cells

RBL-2H3 mast cells were used as an *in vitro* model for evaluating the anti-allergic effect of steptochlorin and for elucidating its molecular mechanisms. As a first step, the cytotoxicity of streptochlorin was measured by the MTT-based viability assay. RBL-2H3 cells were treated with various concentrations of streptochlorin for 24 h. Streptochlorin concentrations of up to 100 µM did not appreciably affect the viability of RBL-2H3 cells (Fig. 1B).

Secretion of preformed allergic mediators, such as histamine and various proteases, in granules by mast cells are a key step in local allergic reactions [13]. The measurement of released β-hexosaminidase from cells has been used as an indicator of mast cell degranulation [14]. Thus, we tested whether streptochlorin inhibited the antigen-stimulated degranulation in RBL-2H3 cells. There was a significant and dose-dependent inhibition of DNP-HSA-induced degranulation in RBL-2H3 cells (Fig. 1C).

### Streptochlorin suppresses expression and secretion of allergic and pro-inflammatory cytokines

**Figure 2:**
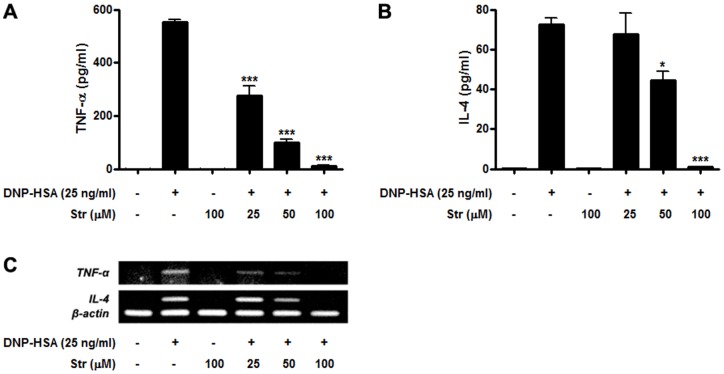
Effect of streptochlorin on the expressional levels of TNF-α and IL-4 in RBL-2H3 cells. The levels of TNF-α (A) and IL-4 (B) in the media were assayed by ELISA. Total mRNA expression levels of TNF-α and IL-4 were measured by semiquantitative RT-PCR (C). β-actin was used as an internal control. The results are expressed as mean ± standard error of the mean (*n* = 3) for three independent experiments by one-way analysis of variance followed by Dunnett's multiple comparison test. * *P*<0.05 and *** *P*<0.001 significant difference compared with the DNP-HSA only treated group. Str, streptochlorin.

Several cytokines, including TNF-α and IL-4, are crucial for inducing delayed type hypersensitive allergic and inflammatory responses [3], [15]. Therefore, we examined whether streptochlorin could inhibit the release of TNF-α and IL-4 in antigen-induced RBL-2H3 cells. Streptochlorin dose dependently suppressed DNP-HSA-stimulated secretion of TNF-α and IL-4 from RBL-2H3 cells (Fig. 2A and 2B) and the expression levels of the corresponding mRNA (Fig. 2C). These results suggested that streptochlorin inhibits the production of TNF-α and IL-4 by blocking their transcription in FcεRI-stimulated RBL-2H3 cells. Both protein and mRNA levels of TNF-α were significantly reduced by streptochlorin, but those of IL-4 were not statistically significant. These results suggest that although the early receptor-proximal signaling events with Syk activation were inhibited, different molecular engagement such as transcription blocking or increased turnover/instability of the mRNA molecules for TNF-α and IL-4 expression or downstream signaling events could be involved.

### Streptochlorin inhibits phosphorylation of Syk kinase in RBL-2H3 cells

**Figure 3:**
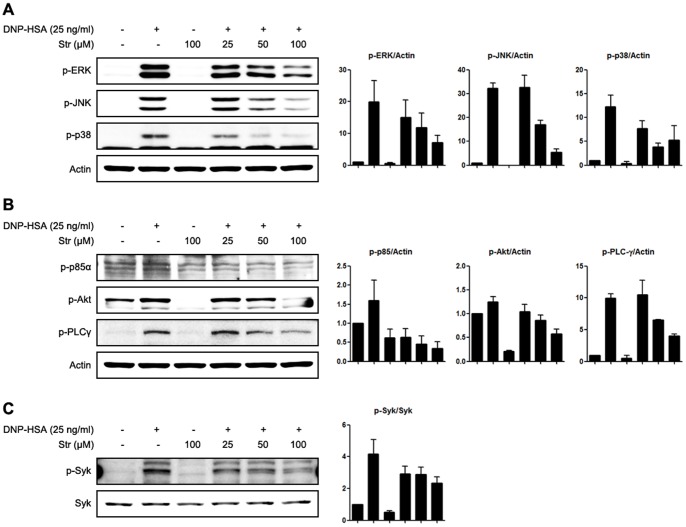
The effect of streptochlorin on MAPKs, PI3K, and Syk signaling in RBL-2H3 cells. IgE-primed RBL-2H3 cells were stimulated with 25 ng/mL of DNP-HSA with or without streptochlorin at the indicated doses. Phosphorylated MAPKs (A), PI3K (B), and Syk (C) were detected by Western blot analysis. β-actin was used as the internal control. Right panels are histograms of densitometric analysis from three independent Western blots. Str, streptochlorin.

The aggregation of antigen, IgE, and FcεRI induces various intracellular signaling pathways in mast cells [3], [5]. We investigated potential signaling targets responsible for the inhibitory actions of streptochlorin on mast cells. First, the activating phosphorylation of mitogen-activated protein kinases (MAPKs) was examined. Streptochlorin suppressed the phosphorylation of major MAPKs, especially those associated with expression of a variety of cytokines, such as p38, ERK 1/2, and JNK (Fig. 3A). Further, we assessed the effect of streptochlorin on phosphatidylinositol-3 kinase (PI3K) signaling, which mediates upstream signaling of MAPKs that precede degranulation [3], [5]. Phosphorylation of p85α, the PI3K subunit, was inhibited by streptochlorin and led to a strong reduction in the phosphorylation of downstream signaling molecules, such as Akt and phospholipase C-γ (PLC-γ) (Fig. 3B). Finally, we verified that streptochlorin suppressed the phosphorylation of Syk kinase, an upstream signaling molecule of PI3K (Fig. 3C). These data suggest that streptochlorin inhibits degranulation and secretion of cytokines in allergic antigen-induced mast cells by attenuating the Syk kinase signaling pathway.

### Streptochlorin suppresses activation of Lyn and Fyn kinases

**Figure 4:**
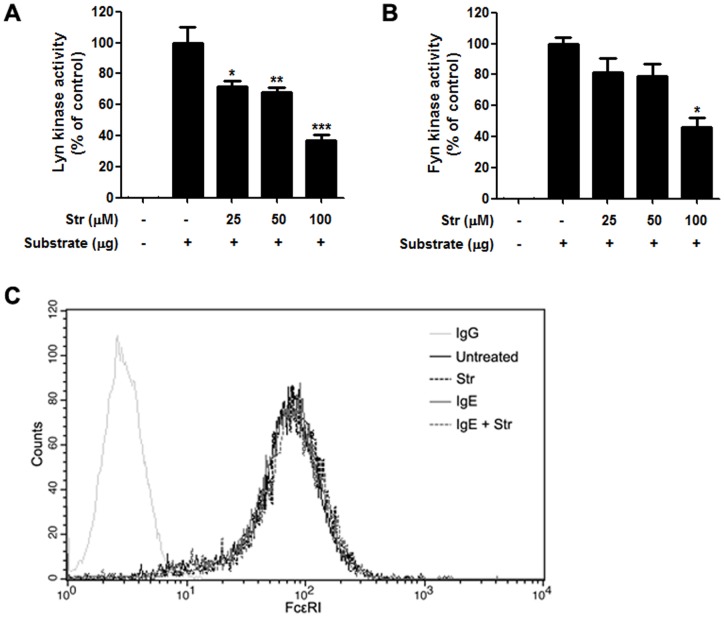
Effect of streptochlorin on expression of FcεRI in RBL-2H3 cells and *in vitro* activity of Lyn and Fyn. The activity of Lyn (A) and Fyn (B) was measured by the ADP-Glo™ kinase assay. The values indicate mean ± standard error of the mean from three independent experiments by one-way analysis of variance followed by Dunnett's multiple comparison test. * *P*<0.05, ** *P*<0.01, and *** *P*<0.001 significant difference from the substrate-alone group. (C) RBL-2H3 cells were treated with or without IgE and streptochlorin for 1 h. The cells were then collected and analyzed by flow cytometry for expression of FcεRI. Representative images are shown from three independent experiments. Str, streptochlorin.

Ag-mediated aggregation of IgE-occupied FcεRI on the mast cell surface leads to activation of the Src family kinases. This in turn phosphorylates immunoreceptor tyrosine-based activation motifs (ITAMs), leading to activation of Syk kinase in the early receptor-proximal signaling events [16], [17]. Therefore, we measured the effect of streptochlorin on activity of the Src family kinases Lyn and Fyn by using an *in vitro* enzyme system. Streptochlorin significantly inhibited the activation of Lyn and Fyn kinases (Fig. 4A and 4B), indicating that streptochlorin can inhibit the activation of Src family kinases together with Syk kinases. To investigate whether the inhibition of Src family kinase activity was caused by interruption of FcεRI expression, we examined the expression of FcεRI on the surface of RBL-2H3 cells treated with or without IgE in the absence or presence of streptochlorin. We found that FcεRI expression was not affected by streptochlorin (Fig. 4C).

### Streptochlorin suppresses DNFB -induced allergic dermatitis model

**Figure 5:**
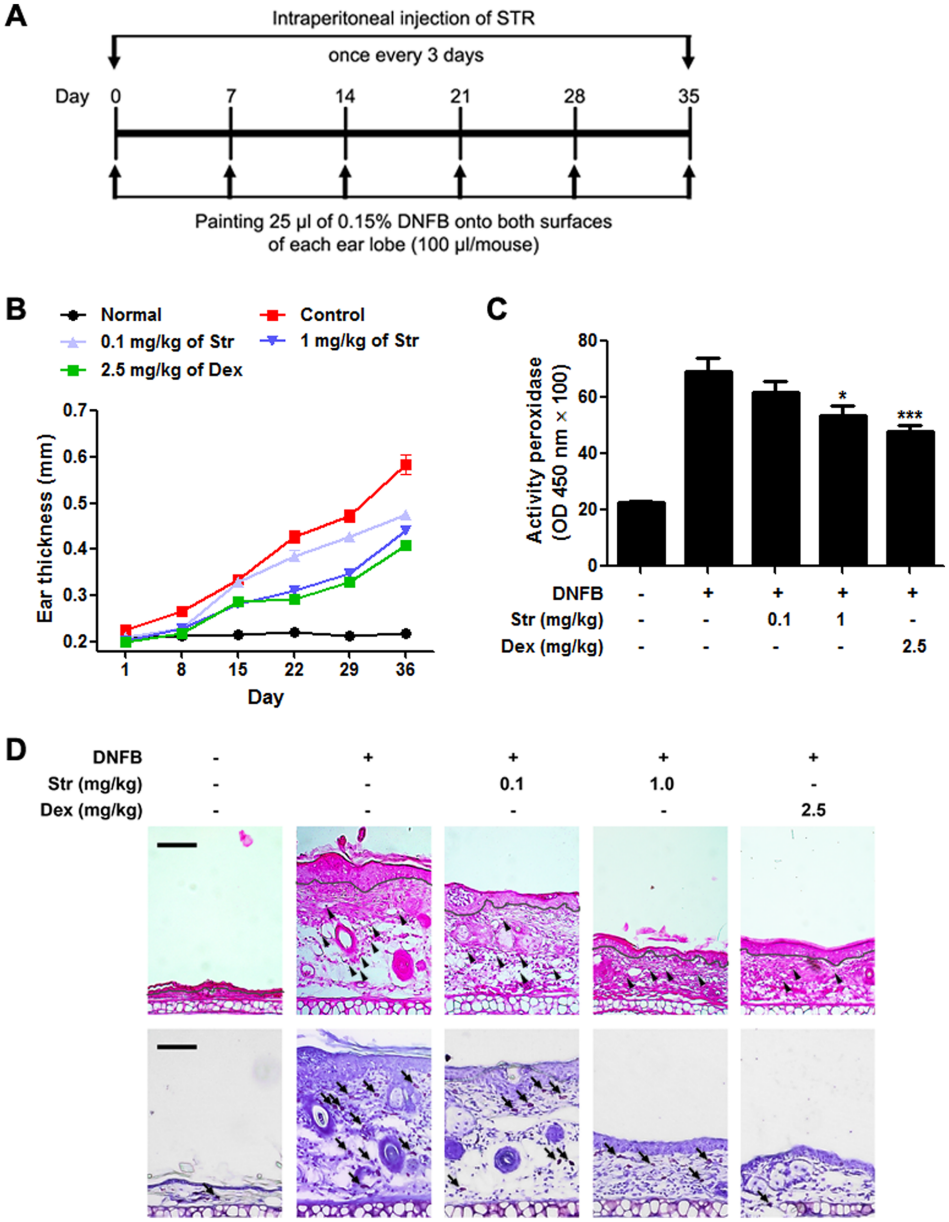
The effect of streptochlorin on DNFB-induced allergic dermatitis. (A) The experimental scheme. (B) Balb/c mice (*n* = 5) were sensitized with DNFB with the indicated amount of streptochlorin. Ear thickness was determined on the indicated dates. (C) Ear lobe tissue homogenates were prepared from mice in each group, and the lysates were measured by an MPO assay. (D) Histologic images of the ear lobes of mice in each group stained with hematoxylin (upper panel) and toluidine blue (lower panel) are shown. Dexamethasone was used as a positive control. The gray line means a boundary of dermis and epidermis, and infiltrated immune cells (arrowhead) and mast cells (arrow) were indicated. The values indicate mean ± standard error of the mean from three independent experiments by one-way analysis of variance followed by Dunnett's multiple comparison tests. * *P*<0.05 and *** *P*<0.001 significant difference from DNFB only treated group. Str, streptochlorin; Dex, dexamethasone.

DNFB can induce contact allergic dermatitis by hypersensitivity [18]. Therefore, we observed the attenuation of hypersensitive reactions after treatment with streptochlorin in the DNFB-induced allergic dermatitis mouse model (Fig. 5A). The streptochlorin-treated group showed a significant decrease in swelling and thickness of the ear lobes compared to the DNFB-alone-treated control group (Fig. 5B). Decrease in scratching behavior in mice was also observed (data not shown). Measurement of MPO, the most abundant peroxidase enzyme in granulocytes, revealed that administration of streptochlorin suppressed the enzyme activity in a dose-dependent manner (Fig. 5C). Further, histological examination of the inflamed ear lobes by hematoxylin and eosin staining were observed. Administration of streptochlorin reduced swelling of the ear lobes and recruitment of inflammatory cells into the inflamed ear lobes, suggesting that granulocytes, including mast cells, eosinophils, and neutrophils, were less likely to be recruited into the inflamed site upon treatment with streptochlorin (Fig. 5D).

### Streptochlorin modulates production and secretion of pro-inflammatory cytokines and IgE in a DNFB-induced allergic dermatitis model

**Figure 6:**
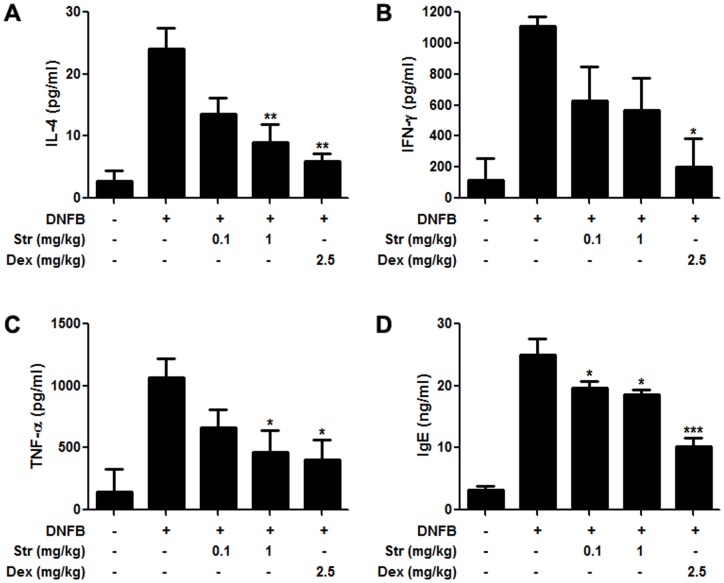
Effect of streptochlorin on cytokines in inflamed ear lobes and serum IgE in DNFB-induced allergic dermatitis. Production of IL-4 (A), IFN-γ (B), and TNF-α (C) was measured with ear lobe lysates. IgE was measured with serum samples (D). Dexamethasone was used as a positive control. The obtained values for IL-4, IFN-γ, and TNF-α were normalized to the β- actin which is used as an internal control. The values indicate mean ± standard error of the mean from three independent experiments by one-way analysis of variance followed by Dunnett's multiple comparison tests. * *P*<0.05, ** *P*<0.01, and *** *P*<0.001 significant difference from DNFB only treated group. Str, streptochlorin; Dex, dexamethasone.

Contact hypersensitivity is caused by cytokines as well as infiltrating inflammatory cells [19]. We investigated the anti-allergic activity of streptochlorin by monitoring the production of allergy-associated cytokines, such as IL-4, INF-γ, and TNF-α, in the ear lobes after DNFB treatment. Production of IL-4 (Fig. 6A), INF-γ (Fig. 6B), and TNF-α (Fig. 6C) was inhibited by streptochlorin in a dose-dependent manner. In addition, we examined levels of serum IgE, which plays a critical role in the development of allergic reactions [15]. The production of IgE was also inhibited in a dose-dependent manner after administration of streptochlorin (Fig. 6D).

### Streptochlorin suppresses PCA-induced allergic dermatitis model

**Figure 7:**
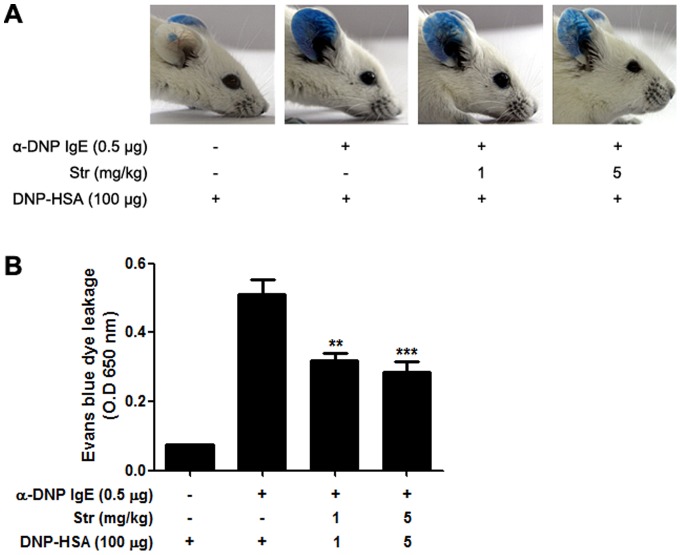
Effects of streptochlorin on IgE-mediated PCA reaction in mice. Upon antigen challenge, leakage of injected Evans blue into the ear was significantly reduced in streptochlorin-injected (i.v.) mice. Representative pictures of the ear are shown (A). Vascular leakage assayed by Evans blue extravasation into ear tissue. The same size ears were excised 20 min after i.v. challenge with antigen and Evans blue and subsequently extracted in formamide (B). Each column shows mean optical density at 650 nm. The values indicate mean ± standard error of the mean (*n* = 3), from three independent experiments by one-way analysis of variance followed by Dunnett's multiple comparison test. ** *P*<0.01 and *** *P*<0.001 significantly different when compared with α-DNP IgE and DNP-HSA treated groups. Str, streptochlorin.

To evaluate the anti-allergic activity of streptochlorin *in vivo*, the effect of streptochlorin on DNP/IgE-induced PCA was examined using a mouse model. Compared to the normal mice group, intradermal administration of anti-DNP-IgE induced a prominent enhancement of Evans blue dye leakage into the ears of control mice. Leakage of injected Evans blue dye was significantly suppressed by streptochlorin pretreatment (Fig. 7A and 7B). These data suggest that streptochlorin potentially inhibited allergic responses *in vivo* not only by regulating mast cells activation in tissues but also by regulating adaptive immune cells in chronic allergic inflammation. No major side-effects were observed with tested doses of streptochlorin treatment including the body weight loss. However, i*n vivo* animal studies should be conducted to determine whether high concentrations of streptochlorin have any untoward side effects.

### Streptochlorin can affect T-cell activation, but not T-cell development

**Figure 8:**
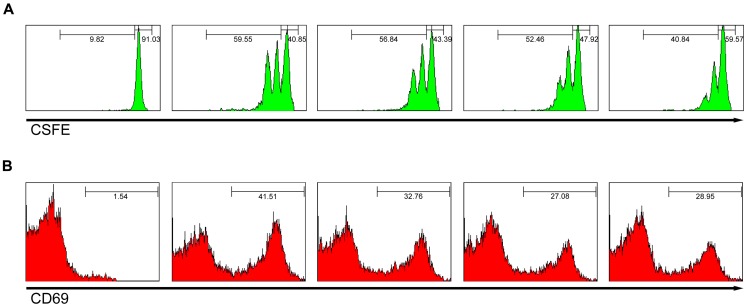
Effect of streptochlorin on T-cell proliferation and activation *in vivo*. (A) Stimulated lymph node T-cells by anti-CD3/anti-CD28 on plates were analyzed by flow cytometric CFSE dilution for proliferation assay at day 2 after treatment with streptochlorin. (B) CD69 expression level for early T-cell activation was analyzed by flow cytometer at day 1 after treatment *in vivo* with streptochlorin. PE conjugate anti-CD69 was purchased from BD Biosciences.

It might be predicted that inhibition of Lyn and Fyn kinases can adversely affect T-cell activation and consequently affect host defense. In our study, administration of streptochlorin to mouse did not show any significant effect on T-cell activation. However dexamethasone decreased the weight of lymph nodes and thymocytes (Fig. S1). Further, the population of CD3^+^ T cells in lymph nodes and CD4^+^/CD8^+^ T cells in thymus was not affected by streptochlorin administration when compared with dexamethasone which decreased the T-cell population in lymph nodes and thymus. Furthermore, streptochlorin slightly suppressed CD3-stimulated T-cell division and the expression of CD69. The activation markers of CD3-stimulated T cells were also decreased dose-dependently by streptochlorin (Fig. 8). Taken together, we suggest that streptochlorin can affect T-cell activation *in vitro* through attenuation of activation signal mediators such as Src kinases. However, streptochlorin did not show any prominent effect on T-cell development *in vivo*.

## Discussion

Allergic reactions are induced by the release of allergic inflammatory mediators and various pro-inflammatory cytokines from mast cells. The activation of mast cells depends initially on the crosslinking of IgE and FcεRI, and this high-affinity interaction provides strong intracellular signals leading to secretion of histamine, proteases, leukotrienes, and cytokines [15]. The products of mast cells have many pathologic and physiological roles in various tissues and can cause allergic inflammation followed by allergic diseases.

Upon initiation of FcεRI signaling, Src kinases Lyn and Fyn interact with the intracellular portion of FcεRI, subsequently activating Syk and other tyrosine kinases. Syk kinase is a key player in mast cell activation because it leads to the activation of several downstream signaling molecules, including PI3K, Akt, PLCγ, and major MAPKs [20], [21]. Activation of the signaling cascade regulates the degranulation and production of allergic and inflammatory cytokines [20]. Therefore, Syk kinase is an attractive target for therapeutic inhibitors of mast cell-related allergic diseases. It might be predicted that inhibition of Src kinases can adversely affect T-cell activation and consequently affect host defense. Earlier studies revealed that the activation of mast cells is initially dependent on the interaction of FcεRI with the Src kinases Lyn and Fyn and subsequently on the downstream activation of Syk and other tyrosine kinases [16]. However, Lyn also exhibits a negative role and this is the predominant feature of Lyn in early signaling events. In contrast, Fyn is exclusively a positive regulator in mast cells [16], [22]. Studies on *in vivo* knock out models about the role of Fyn and Lyn revealed that Ca^2+^ signaling is inhibited in Lyn-deficient cells, whereas degranulation which is normally activated by Fyn, is increased, indicating a negative effect of the Lyn pathway on the Fyn signaling. The crosstalk between the two pathways was also suggested [5]. However, in our present study, streptochlorin suppressed both Lyn and Fyn phosphorylation in antigen-stimulated cells. Our results are in agreement with earlier report that carotenoids significantly inhibited the levels of both phosphorylated Lyn and Fyn when compared to the control cells indicating the role of carotenoids in inhibiting antigen-induced aggregation of FcεRI-mediated degranulation signals owing to their potential use in inflammatory and allergic reactions [23]. Several drugs having inhibitory effects on tyrosine kinases are now approved for anticancer therapy. Since imatinib has been approved in the USA and Europe for chronic phase myelogenous leukemia, tyrosine kinase inhibitors such as dasatinib, bosutinib and gefitinib have emerged as a new class of anticancer agents with proven efficacy in several types of carcinoma [24], [25]. Likewise, streptochlorin having capability of tyrosine kinase inhibitor and can be used as a therapeutic strategy for the treatment of allergic dermatitis. Currently several compounds that inhibit the activation of Syk kinase in mast cells are under clinical trials [4], [26]. In addition to regulating mast cell activation, streptochlorin also inhibited nitric oxide and cyclooxygenase-2 expression in macrophages (data not shown). Furthermore, indole-3-carbinol which has structural similarity to streptochlorin has been reported to have anti-inflammatory effects [27], but not on anti-allergic effect.

In conclusion, the present study revealed for the first time that streptochlorin possesses anti-allergic properties. The strong synergistic effect exhibited by streptochlorin via regulation of multiple signaling pathways such as Lyn/Fyn and Syk activation might be responsible for its potent anti-allergic effects.

## Supporting Information

Figure S1 (TIF)

Materials and Methods S1 (DOCX)
