# Supplementary figures and images for: Streptochlorin Suppresses Allergic Dermatitis and Mast Cell Activation via Regulation of Lyn/Fyn and Syk Signaling Pathways in Cellular and Mouse Models
Source: PLoS One. 2013 Sep 27;8(9):e74194. doi: 10.1371/journal.pone.0074194 (PMC3785495; doi:10.1371/journal.pone.0074194)

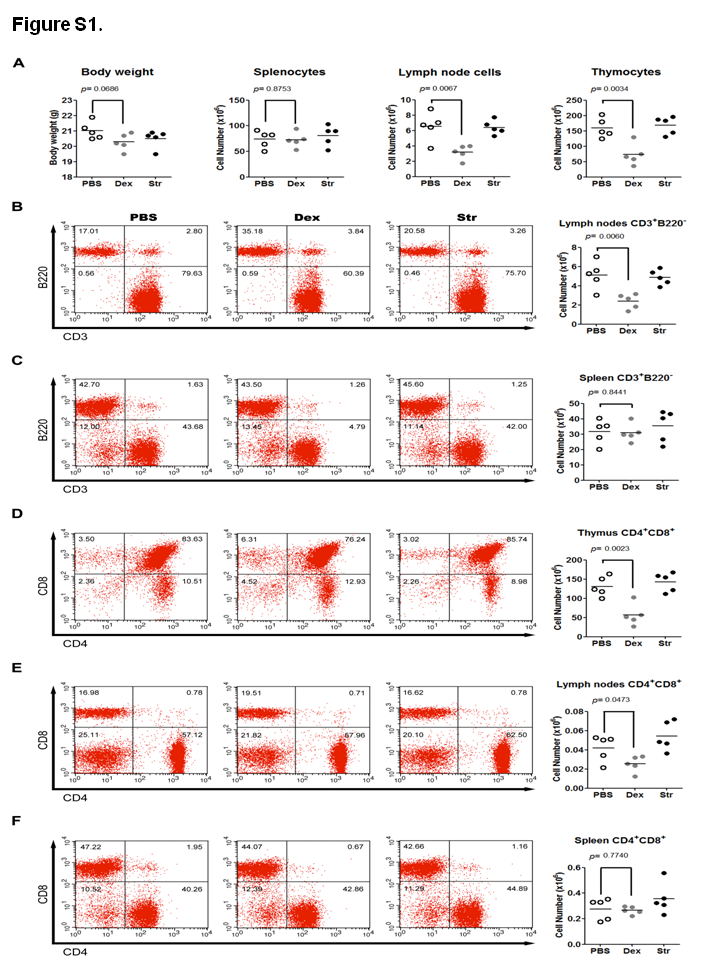

Supplement: Figure S1 — (TIF) [file pone.0074194.s001.tif]
