# Supplementary material for: Streptochlorin Suppresses Allergic Dermatitis and Mast Cell Activation via Regulation of Lyn/Fyn and Syk Signaling Pathways in Cellular and Mouse Models
Source: PLoS One. 2013 Sep 27;8(9):e74194. doi: 10.1371/journal.pone.0074194 (PMC3785495; doi:10.1371/journal.pone.0074194)
Supplement: Materials and Methods S1 — (DOCX) [file pone.0074194.s002.docx]

**Supporting Information**

**Materials and Methods**

Effect of streptochlorin on T-cell development

Dexamethasone (2.5 mg/kg) or streptochlorin (1 mg/kg) was provided three times every 3 days, and mice were sacrificed on day 9. Lymph node cells, splenocytes, and thymocytes were stained with indicated antibodies and analyzed by FACSCalibur. Anti-CD4, anti-CD8, anti-B220 and anti-CD3 antibodies were purchased from BD Biosciences.

Preparation of cells

Lymph nodes, spleens, and thymus were collected and processed. Cell suspensions were obtained by FACS buffer (PBS containing 5% FBS). RBCs were lysed using ACK buffer (150 mM NH_4_Cl, 10 mM KHCO_3_) for 30 sec at room temperature, and cells were resuspended in FACS buffer. Viable cells were counted with a hemocytometer and trypan blue exclusion.
